# Polyunsaturated fatty acyl-coenzyme As are inhibitors of cholesterol biosynthesis in zebrafish and mice

**DOI:** 10.1242/dmm.013425

**Published:** 2013-09-18

**Authors:** Santhosh Karanth, Vy My Tran, Balagurunathan Kuberan, Amnon Schlegel

**Affiliations:** 1 University of Utah Molecular Medicine (U2M2) Program, University of Utah School of Medicine, Salt Lake City, UT 84112, USA; 2 Department of Internal Medicine, Division of Endocrinology, Metabolism and Diabetes, University of Utah School of Medicine, Salt Lake City, UT 84112, USA; 3 Department of Bioengineering, University of Utah, Salt Lake City, UT 84112, USA; 4 Department of Medicinal Chemistry, College of Pharmacy, University of Utah, Salt Lake City, UT 84112, USA; 5 Department of Biochemistry, University of Utah School of Medicine, Salt Lake City, UT 84112, USA

## Abstract

Lipid disorders pose therapeutic challenges. Previously we discovered that mutation of the hepatocyte β-hydroxybutyrate transporter Slc16a6a in zebrafish causes hepatic steatosis during fasting, marked by increased hepatic triacylglycerol, but not cholesterol. This selective diversion of trapped ketogenic carbon atoms is surprising because acetate and acetoacetate can exit mitochondria and can be incorporated into both fatty acids and cholesterol in normal hepatocytes. To elucidate the mechanism of this selective diversion of carbon atoms to fatty acids, we fed wild-type and *slc16a6a* mutant animals high-protein ketogenic diets. We find that *slc16a6a* mutants have decreased activity of the rate-limiting enzyme of cholesterol biosynthesis, 3-hydroxy-3-methylglutaryl-coenzyme A reductase (Hmgcr), despite increased Hmgcr protein abundance and relative incorporation of mevalonate into cholesterol. These observations suggest the presence of an endogenous Hmgcr inhibitor. We took a candidate approach to identify such inhibitors. First, we found that mutant livers accumulate multiple polyunsaturated fatty acids (PUFAs) and PUFA-CoAs, and we showed that human HMGCR is inhibited by PUFA-CoAs *in vitro*. Second, we injected mice with an ethyl ester of the PUFA eicosapentaenoic acid and observed an acute decrease in hepatic Hmgcr activity, without alteration in Hmgcr protein abundance. These results elucidate a mechanism for PUFA-mediated cholesterol lowering through direct inhibition of Hmgcr.

## INTRODUCTION

Atherosclerotic cardiovascular disease is the leading cause of death worldwide (Lozano et al., 2012). Elevated serum cholesterol is a causal risk factor for the development and progression of atherosclerosis (Kannel et al., 1964). The most widely used treatment for atherosclerosis is statin drugs, which potently inhibit 3-hydroxy-3-methylglutaryl-coenzyme A reductase (HMGCR) and dramatically lower cardiovascular mortality (Baigent et al., 2010). Nevertheless, a substantial fraction of people receiving statin drugs do not tolerate them, with vague muscle aches being the main cause of drug discontinuation (Reinhart and Woods, 2012). Hence, there is considerable interest in identifying alternative strategies for lowering serum cholesterol, including life-style changes, micronutrient supplementation and pharmaceutical development.

A greater understanding of the underlying factors regulating lipid metabolism holds the promise of yielding novel therapies. The zebrafish has numerous technical advantages for modeling human diseases (Lieschke and Currie, 2007), and is emerging as a major resource for studying lipid metabolism (Schlegel, 2012; Sadler et al., 2013). First, the zebrafish utilizes a lipid packaging machinery that is conserved among metazoans to transport neutral lipids from their sites of absorption (intestine and yolk) or synthesis (liver) to peripheral organs. The molecular players involved include apolipoproteins (Babin et al., 1997) and apolipoprotein processing enzymes (Marza et al., 2005; Schlegel and Stainier, 2006). Following release into the circulation, lipoproteins are modified in zebrafish blood by a machinery that is also highly conserved in evolution. For instance, zebrafish carry an ortholog of the human cholesteryl ester transfer protein (*CETP*) gene. A *CETP* ortholog is frustratingly absent in commonly used rodent models (Haa and Barter, 1982), rendering the study of atherosclerosis difficult: rodents are inherently resistant to atherosclerosis (Yin et al., 2012), and background manipulations such as targeted deletion of the *Apoe* or *Ldlr* genes are required to drive atherosclerosis. Unlike mice and rats, an ortholog of the human *CETP* gene is retained in zebrafish, causing the circulating lipoprotein particles to resemble human lipoprotein particles in abundance and composition; this conserved lipoprotein metabolism contributes to the susceptibility of zebrafish to atherosclerosis when placed on a high-cholesterol diet (Stoletov et al., 2009). Furthermore, the deposition of sub-intimal cholesterol can be monitored in real time, in live animals (Fang et al., 2011). In addition to their genetic propensity to developing atherosclerosis when placed on high-cholesterol diets, zebrafish develop obesity, hypertriglyceridemia, hepatic steatosis and characteristic adipocyte gene expression changes when over-fed their normal diet (Oka et al., 2010). These effects can be reversed by caloric restriction.

In a forward genetic screen for zebrafish mutants with lipid storage defects, we discovered that the liver has a dedicated β-hydroxybutyrate transporter whose loss causes nutritionally suppressible hepatic steatosis (Hugo et al., 2012). This transporter normally exports the major ketone bodies produced by the liver under starvation conditions, acetoacetate and β-hydroxybutyrate, which are partially oxidized short-chain fatty acid catabolites of longer-chain fatty acids and certain amino acids. Together, these ketone bodies are the major fuel of fasting, providing energy to the brain when glucose is depleted (Cahill, 2006). When administered radiolabeled leucine, a ketogenic amino acid, never-fed zebrafish *slc16a6a* mutant larvae divert the trapped ketogenic carbon atoms into triacylglycerol exclusively (Hugo et al., 2012). This selectivity is unanticipated because carbon atoms from mitochondrial leucine catabolism can be funneled to cytoplasmic cholesterol. Specifically, the acetate and acetoacetate generated from leucine breakdown can exit mitochondria and be used to synthesize both fatty acids and cholesterol (Mathias et al., 1981).

**TRANSLATIONAL IMPACT**

**Clinical issue**

Cardiovascular disease is the leading cause of death worldwide. Elevated blood cholesterol, which can result in atherosclerotic plaques, is a major risk factor in the development of heart disease; thereby, research has focused on targeting this aspect of disease pathophysiology. Globally, the most widely prescribed drugs are statins, which lower serum cholesterol by inhibiting the rate-limiting step of cholesterol synthesis catalyzed by 3-hydroxy-3-methylglutaryl-coenzyme A reductase (HMGCR). Although the use of statins has reduced cardiovascular disease mortality rates, many individuals who would benefit from lipid lowering cannot tolerate the drugs because of unpleasant side effects. Notably, the occurrence of muscle aches has been reported in nearly one fifth of people receiving statins. Lowering the dose or switching from one statin to another often fails to alleviate symptoms; therefore, identifying alternative approaches for lowering cholesterol is a priority in cardiovascular disease research.

**Results**

In this study, the authors exploit the zebrafish model to identify novel strategies for lowering blood cholesterol. They previously identified a zebrafish mutant that fails to release ketone bodies (the major fuel during conditions of starvation) from the liver and develops hepatic steatosis. In this mutant, which lacks a key liver transporter, trapped ketogenic amino acids are selectively diverted to triacylglycerol synthesis instead of cholesterol synthesis. Here, the authors show that, in addition to triacylglycerol, polyunsaturated fatty acids accumulate in the livers of these zebrafish mutants. Furthermore, mutant livers display decreased Hmgcr activity, despite an increase in abundance of this protein. The group demonstrates that coenzyme-A-activated polyunsaturated fatty acids (PUFA-CoAs) inhibit human HMGCR *in vitro*, suggesting that these molecules act as an endogenous inhibitor of the enzyme. This conclusion is supported by subsequent *in vivo* experiments in mice, in which injection of PUFA-CoAs resulted in an acute decrease in Hmgcr activity without affecting protein abundance.

**Implications and future directions**

This study provides physiological, biochemical and pharmacological evidence that PUFA-CoAs act as competitive inhibitors of HMGCR and thereby cholesterol synthesis. In addition to revealing a new mechanism by which polyunsaturated fatty acids can regulate lipid biogenesis in vertebrates, the work reveals a new therapeutic candidate for the treatment of cardiovascular disease. Polyunsaturated fatty acids are available in pharmaceutical preparations (fish oil supplements) for lowering serum triglycerides, and future work should focus on testing whether doses of fish oils could be fine-tuned to be used as adjuncts to statins.

Here we identify a molecular mechanism for this selective diversion of carbon atoms to fatty acyl chains and not to cholesterol that has broad implications for the pharmacological suppression of cholesterol synthesis. We trigger severe hepatic steatosis in *slc16a6a* mutants by feeding them ketogenic diets, and find that, in addition to triacylglycerol, polyunsaturated fatty acid (PUFA)-CoAs (activated intermediates of neutral and phospholipid synthesis) accumulate in the mutants’ livers. Furthermore, PUFA-CoAs are competitive inhibitors of Hmgcr *in vitro*, with physiologically plausible kinetic properties. Finally, short-term administration of a high dose of a single PUFA ethyl ester to mice decreases liver Hmgcr activity, without altering Hmgcr protein abundance. Chronic administration of PUFAs to mice disrupts the maturation of sterol-regulatory element binding proteins (SREPBs), the master transcription factors controlling lipid synthesis, and indirectly lowers expression of several genes encoding enzymes involved in fat and cholesterol production (Worgall et al., 1998; Mater et al., 1999; Xu et al., 1999; Yahagi et al., 1999). Furthermore, chronic PUFA supplementation can ameliorate hepatic steatosis in *ob*/*ob* mice through suppressing Srebp1 (Sekiya et al., 2003). Our findings indicate that PUFACoAs (specifically) also have a direct inhibitory effect on Hmgcr activity. These findings elucidate a newly identified beneficial role to high dose fish oil supplementation.

## RESULTS

### Ketogenic diets cause massive hepatic steatosis in *slc16a6a* mutants

In order to elucidate a molecular mechanism for the selective diversion of ketogenic carbon atoms that we previously observed in zebrafish larvae, we prepared two isocaloric, isoproteic diets that differ in fat content (4% versus 12%, hereafter ‘K4’ and ‘K12’; supplementary material Table S1) and fed them to juvenile animals. Conventional, commercially available adult feeds have relatively high protein and low fat contents (supplementary material Table S2). The diets we prepared have much higher fiber (as opposed to digestible carbohydrates) than commercial diets. We hypothesized that this novel formulation of high protein and low carbohydrate would be ketogenic, and, when fed to *slc16a6a* mutants, would trigger hepatic steatosis. Furthermore, examining these older, nutritionally sufficient animals would allow us to perform a series of mechanistic experiments that were not feasible in smaller larvae.

**Fig. 1.**
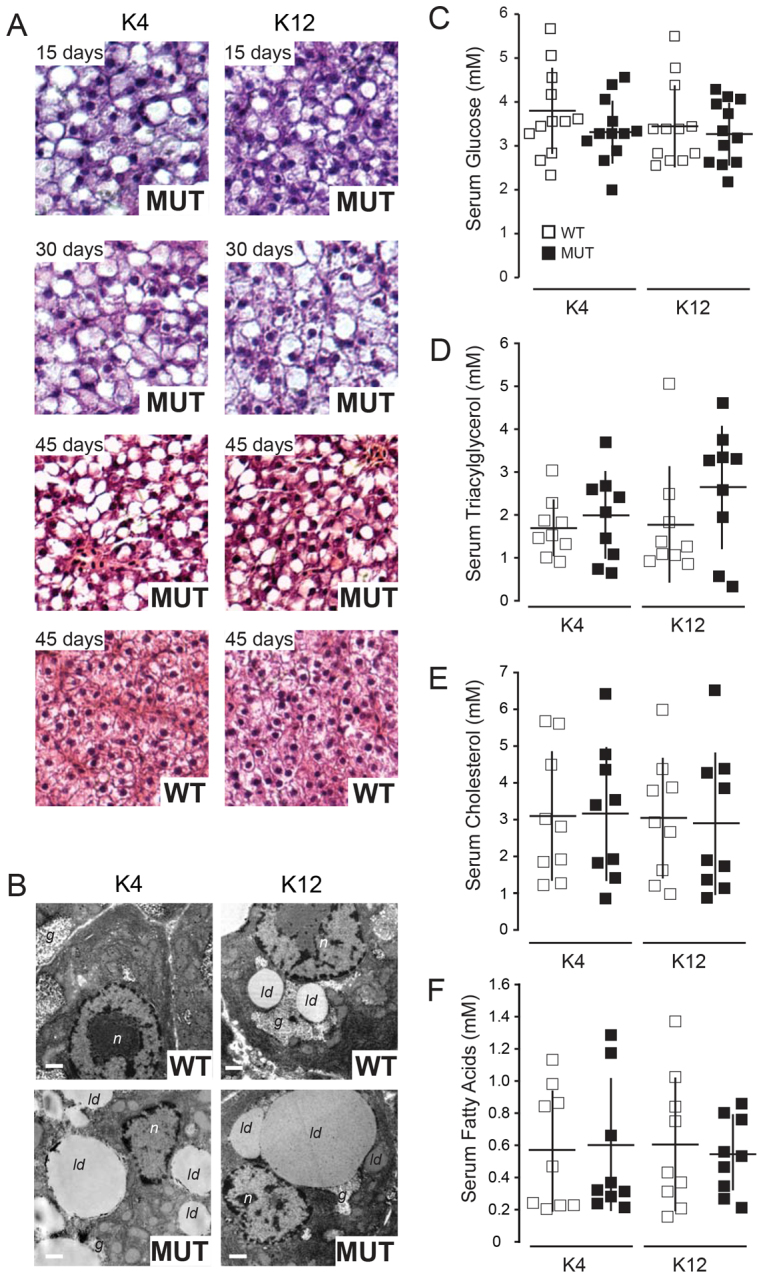
**Ketogenic diets trigger hepatic steatosis in *slc16a6a* mutants.** At 30 dpf, wild-type (WT) and *slc16a6a* mutant (MUT) zebrafish juveniles were placed on ketogenic diets (K4 and K12; supplementary material Table S1). (A) Livers were harvested and analyzed after 15, 30 or 45 days on diets. MUT livers showed progressive steatosis during the study, whereas WT did not. (B) Transmission electron microscopy of liver sections; cytoplasmic lipid droplets (*ld*), nuclei (*n*), glycogen granules (*g*). Scale bars: 1 μm. (C–F) Fasting blood glucose, triacylglycerol, cholesterol (total) and free fatty acids did not differ among the cohorts placed on either ketogenic diet (K4 or K12). All data are mean ± s.d.

Consistent with our previous observations, *slc16a6a* mutants developed massive hepatic steatosis over 45 days on both diets (Fig. 1A). Crucially, this accumulation of cytoplasmic neutral lipid droplets was not accompanied by degenerative changes in hepatocyte architecture (Fig. 1B and supplementary material Fig. S1), unlike what is observed in several other zebrafish mutants with hepatic steatosis (Schlegel, 2012). We were concerned that this short-term ketogenic feeding paradigm might trigger secondary metabolic changes such as hyperglycemia or dyslipidemia that could confound subsequent analysis. More specifically, we wished to avoid triggering an insulin-resistant state that would modify the regulatory apparatus that we wished to uncover in ketogenic states (Jornayvaz et al., 2010). Thus, the 45-day course of high-protein low-carbohydrate feeding did not cause hyperglycemia or dyslipidemia before proceeding to further analysis (Fig. 1C–F). Finally, mutants grew equally well on both diets, and had similar body composition at the conclusion of the feeding period (supplementary material Fig. S2 and Table S3). Taken together, these findings revealed that our experimental design was suitable for establishing a nutrient-sufficient state for exploring selective diversion of ketogenic carbon atoms to triacylglycerol.

**Fig. 2.**
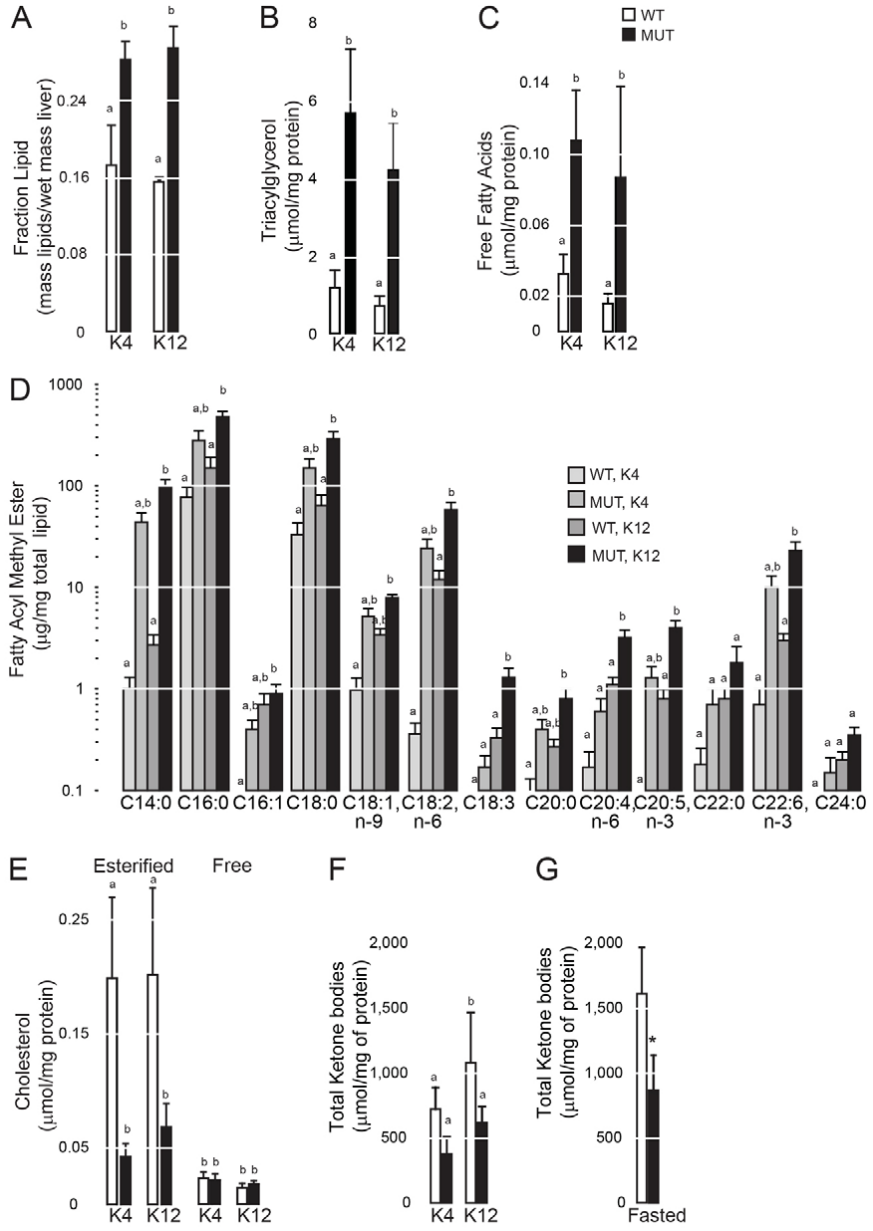
**Triacylglycerol accumulates exclusively in steatotic *slc16a6a* mutants.** (A–F) Lipids were extracted from wild-type (WT) and *slc16a6a* mutant (MUT) livers after 45 days on ketogenic diets (K4 or K12) and analyzed. Results are normalized to liver protein mass. (A) Total lipids (*n*=3). Groups with different letters are statistically different, *P*≤0.005. (B) Triacylglycerol (*n*=5). Groups with different letters are statistically different, *P*≤0.002. (C) Free fatty acids (*n*=5). Groups with different letters are statistically different, *P*≤0.05. (D) Fatty acyl methyl esters (FAMEs) prepared from total lipids (*n*=3). Groups with different letters are statistically different, *P*≤0.05. (E) Cholesteryl esters (*n*=5). Groups with different letters are statistically different, *P*≤0.007. Free cholesterol was not different among the four groups. All data are mean ± s.d. (F) Intrahepatic ketone bodies (*n*=3 per group). Groups with different letters are statistically different, *P*≤0.024. All data are mean ± s.d. and in fasted animals (*n*=5 per group). (G) Intrahepatic ketone bodies were measured in *ad-libitum*-fed and fasted animals. Groups with different letters are statistically different, *P*<0.001. All data are mean ± s.d. Results were compared with one-way ANOVA followed by Tukey’s HSD in A–F, and by two-sided Student’s *t*-test in G.

Measurement of hepatic total lipids, triacylglycerol and free fatty acids after animals were exposed to ketogenic diets revealed that they were all increased in the mutant livers (Fig. 2A–C). Furthermore, several saturated and unsaturated fatty acyl species were increased in whole-lipid extracts of mutant livers (Fig. 2D). Cholesteryl esters were lower and free cholesterol was unchanged (Fig. 2E). Recapitulating our measurement of β-hydroxybutyrate in fasted *slc16a6a* mutants (Hugo et al., 2012), total ketone bodies were lower in mutant livers from animals on either ketogenic diet and in mutants subjected to fasting (Fig. 2F,G).

### Hmgcr is more abundant but less active in *slc16a6a* mutants

**Fig. 3.**
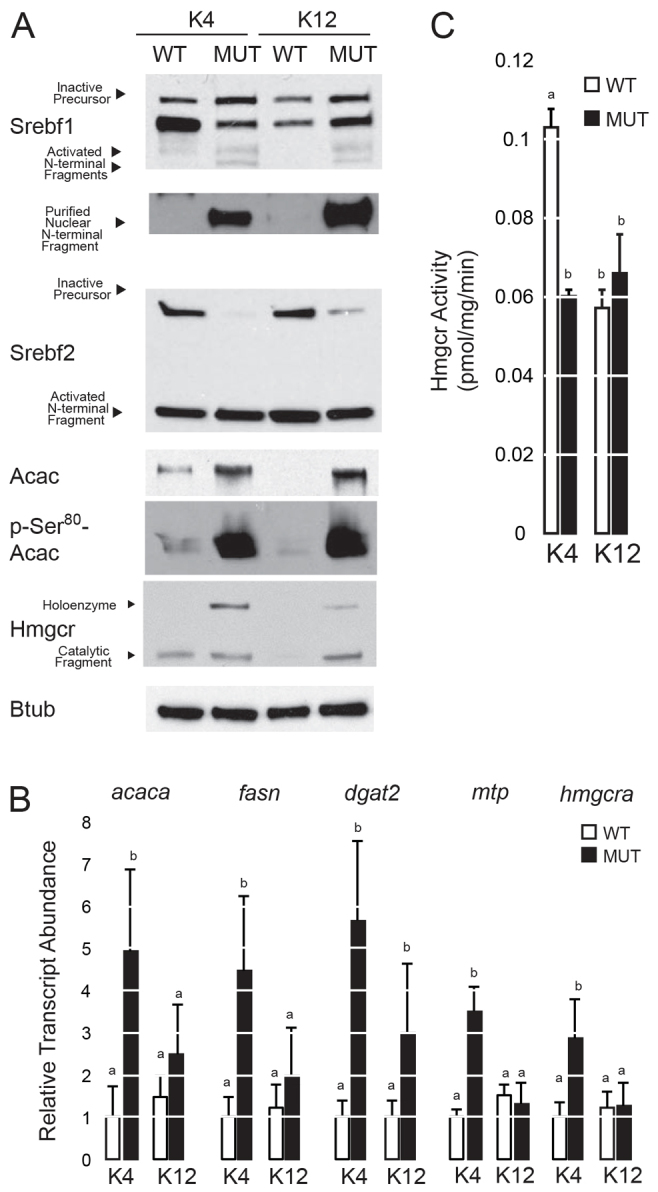
**Activation of Srebf pathways in *slc16a6a* mutant livers.** After 45 days of feeding wild-type (WT) and mutant (MUT) animals ketogenic diets (K4 or K12), livers were harvested and analyzed. (A) Immunoblot analysis of Srebf1, Srebf2, Acac, phospho-Ser80 Acac, Hmgcr and Btub (β-tubulin) (five livers pooled per lane). For Srebf1, an additional nuclear isolation is shown (bottom) because maturation of this transcription factor is not as striking in whole lysates as it is for Srebf2. (B) Abundance of select transcripts encoding enzymes of *de novo* lipogenesis and cholesterol biosynthesis measured by quantitative PCR (*n*=5). Groups with different letters are statistically different, *P*<0.05. Tukey’s HSD. All data are mean ± s.e.m. (C) Hmgcr activity in liver homogenates (*n*=4). Groups with different letters are statistically different, *P*≤0.001. Tukey’s HSD. All data are mean ± s.d.

To understand why *slc16a6a* mutants show increased hepatic accumulation of triacylglycerol only, we assessed the abundance and processing into mature, activated N-terminal fragments of the master transcription factors governing triacylglycerol and cholesterol biosynthesis, Srebf1 and Srebf2, respectively (Jeon and Osborne, 2012). In mutant livers from animals fed either diet, both Srebf proteins showed increased maturation into cleaved N-terminal fragments (Fig. 3A). Wild-type (WT) livers showed appropriate suppression of Srebf2 activation in K12-fed animals. In whole-liver lysates, the degree of Srebf1 processing relative to the total detected was less dramatic than for Srebf2: similar to Srebf1 processing in rat livers, several intermediates of processing and mature fragments of Srebf1 were detected (Owen et al., 2012). In purified nuclei, activated Srebf1 was strikingly increased in *slc16a6a* mutants (Fig. 3A).

Next, we measured the steady-state abundance of Srebf-regulated transcripts encoding crucial enzymes of cholesterol biosynthesis, *de novo* fatty acid synthesis, triacylglycerol production and very-low-density lipoprotein (VLDL) assembly. Mirroring the effect of fasting (Hugo et al., 2012), *slc16a6a* mutants on the K4 diet showed increased expression of several transcripts encoding enzymes of these pathways: *de novo* lipogenesis (*acaca*, *fasn*), triacylglycerol synthesis (*dgat2*) and VLDL assembly (*mtp*) (Fig. 3B). Likewise, the *hmgcra* transcript was induced in *slc16a6a* mutants on the K4 diet. Immunoblot analysis of the rate-limiting enzymes of fatty acid synthesis, acetyl-CoA carboxylase (Acac), and of Hmgcr showed that the K12 diet suppressed Acac and Hmgcr protein levels in WT animals; however, *slc16a6a* mutants showed increased abundance of both proteins on both diets (Fig. 3A). These gene expression and protein abundance results indicated that the increase in triacylglycerol in *slc16a6a* mutant livers might be due, in part, to Acac induction (and of *de novo* lipogenesis, more generally), but the decrease in cholesterol cannot be explained by a simple decrease in Hmgcr protein. Acac serine phosphorylation on Ser80, a post-translational modification catalyzed by AMP-dependent kinase that serves as a feedback control on enzyme activity, was increased in both *slc16a6a* mutant groups (Brownsey et al., 2006). Hmgcr is also phosphorylated by AMP-dependent kinase on the conserved Ser868 residue; however, commercial antibodies fail to detect this site because flanking residues are not sufficiently conserved (supplementary material Fig. S3). Hmgcr phosphorylation is an early event in the feedback inhibition of Hmgcr, exerting a maximal inhibition (30% of control) of this enzyme within 20 minutes of gavage of the product mevalonolactone (Arebalo et al., 1980). Over longer periods of time, Hmgcr phosphorylation seems to be a less significant contributor to alterations in rat cholesterol synthesis than Hmgcr protein abundance (Brown et al., 1979). Because Hmgcr protein was more abundant in both *slc16a6a* groups, we were surprised to observe that, in liver homogenates, Hmgcr activity was 40% lower in *slc16a6a* mutants (Fig. 3C). This reduction in Hmgcr activity was comparable to that seen in WT animals on the K12 diet whose Hmgcr abundance is much lower. These results suggest that severely decreased Hmgcr activity might explain why liver cholesterol levels are not increased in steatotic *slc16a6a* mutants.

**Fig. 4.**
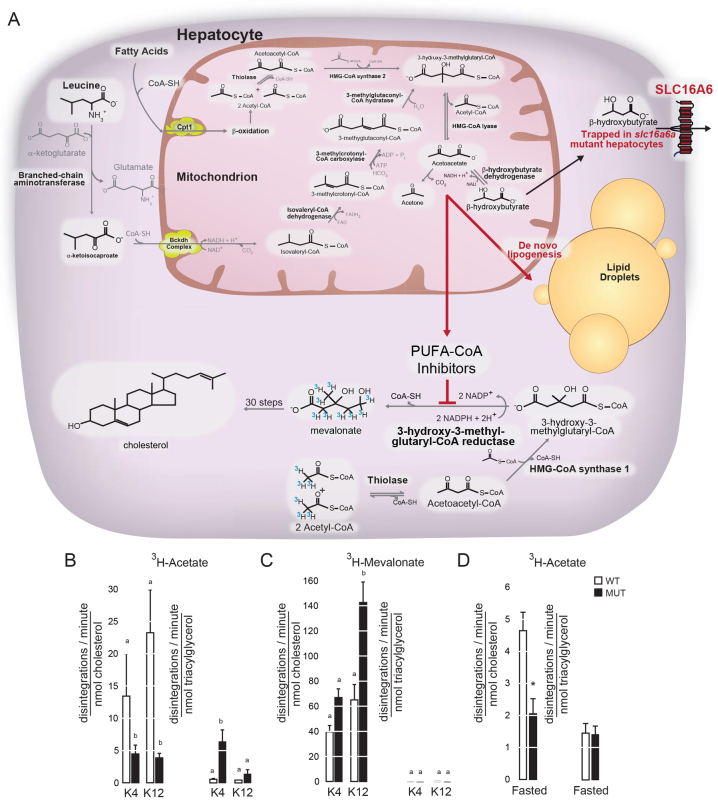
***In vivo* inhibition of Hmgcr in *slc16a6a* mutant livers.** (A) Ketogenesis involves a series of reactions mostly in the mitochondria of hepatocytes, where fatty-acid-derived and select ketogenic-amino-acid-derived carbon atoms are oxidized to acetoacetate. In this scheme, catabolism of acetate (from fatty acids) and the branched-chain amino acid leucine are shown in detail. The terminal product of this pathway is acetoacetate, which can undergo spontaneous decarboxylation to acetone, or be partially reduced to β-hydroxybutyrate. When ketone body export is prevented by mutation of zebrafish *slc16a6a*, the trapped ketone body carbon atoms are diverted to *de novo* lipogenesis, but not to cholesterol. Cholesterol biosynthesis is prevented by inhibition of Hmgcr by the PUFACoAs that accumulate during *de novo* lipogenesis. The ^3^H-marked isotopes injected intraperitoneally were used to address whether there was a block in Hmgcr activity *in vivo*. Note that Hmgcs2 is lacking in zebrafish, eliminating this pathway of acetate consumption. (B,C) Wild-type (WT) and *slc16a6a* mutant (MUT) animals fed ketogenic diets for 45 days were injected intraperitoneally with ^3^H-labeled acetate and ^3^H-labeled mevalonate, and the incorporation of radiotracers into cholesterol and triacylglycerol was measured. Results are reported as disintegrations per minute per nanomole of the indicated lipid. Groups with different letters are statistically different, *P*≤0.05, Duncan’s multiple range test. All data mean ± s.e.m. (*n*=3 per group). (D) Incorporation of radiolabeled acetate into cholesterol and triacylglycerol in WT and MUT animals subjected to a 10-day fast. Groups with different letters are statistically different, *P*≤0.001, two-sided Student’s *t*-test. All data mean ± s.d. (*n*=5 per group).

Decreased Hmgcr activity in the face of increased protein abundance is observed in statin treatment (Brown et al., 1978). To assess whether Hmgcr activity is altered in intact animals, we injected them with labeled acetate and mevalonate and monitored tracer incorporation into triacylglycerol and cholesterol. It is important to note that such a labeling approach in higher vertebrates is fraught with technical pitfalls: the mitochondrial pool of acetate can be syphoned into ketone bodies at a rate greater than the cytoplasmic pool can be incorporated into cholesterol, with dose of tracer, duration of labeling and presence of unlabeled acetate greatly influencing the incorporation of label into cholesterol (Dietschy and McGarry, 1974). Fortunately for us, the mitochondrial HMG-CoA synthase (Hmgcs2) responsible for this diversion of acetate into ketone bodies is only present in birds and higher vertebrates (Fig. 4A) (Boukaftane et al., 1994; Leblanc and Ballantyne, 2000). Indeed, the absence of Hmgcs2 in zebrafish allowed us to perform leucine labeling in fasting to obtain unambiguous evidence that Slc16a6a is a hepatic ketone body transporter (Hugo et al., 2012).

Consistent with the presence of a specific block at the level of Hmgcr, acetate showed greater incorporation into triacylglycerol in mutant livers, whereas mevalonate showed enhanced incorporation into cholesterol (Fig. 4A–C). This latter finding also indicates that the roughly 30 enzymatic steps following Hmgcr in catalyzing cholesterol synthesis are not defective in mutant livers (Brown et al., 1978). To address whether Hmgcr inhibition could be achieved outside our ketogenic dietary model, we subjected animals that were maintained on conventional diets to a fast and assessed the incorporation of radiolabeled acetate into cholesterol and triacylglycerol. Consistent with our results in ketogenic-diet-fed animals, *slc16a6a* mutants showed blunted incorporation of acetate into cholesterol when fasted (Fig. 4D). This result validated our ketogenic dietary paradigm and strongly suggests that Hmgcr activity is inhibited in *slc16a6a* mutant livers even though the protein is more abundant.

### PUFA-CoAs inhibit Hmgcr

**Fig. 5.**
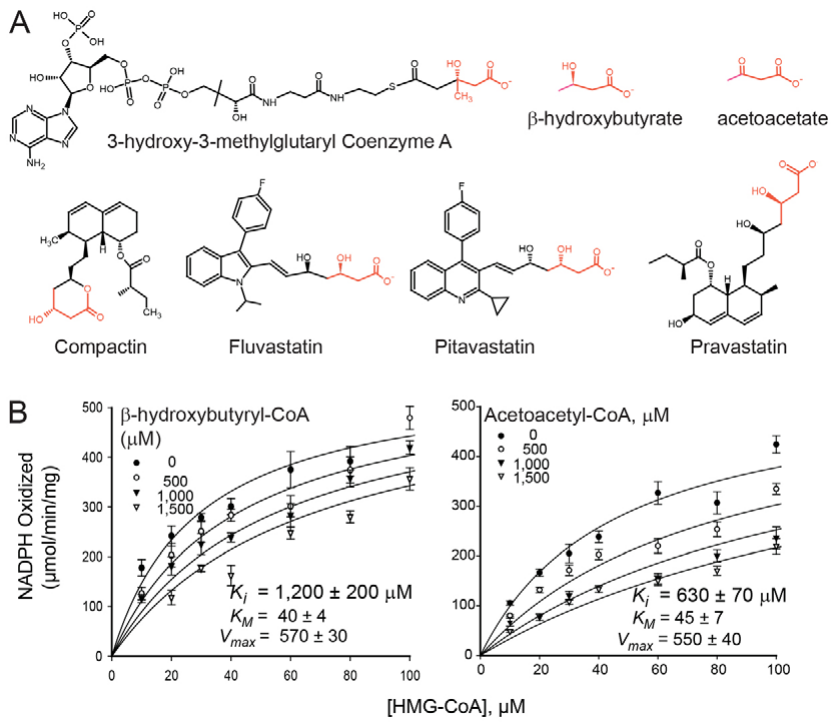
**CoA thioesters of ketone bodies are not plausible inhibitors of HMGCR.** (A) Structure of the HMGCR substrate 3-hydroxy-3-methylglutaryl coenzyme A, the ketone bodies β-hydroxybutyrate and acetoacetate, and four statin inhibitors of HMGCR. The seemingly common pharmacophore is highlighted in red. (B) Michaelis-Menten kinetic analysis of human HMGCR activity with the indicated test inhibitors suggests that neither is physiologically likely: the *K_i_* for both is much higher than the *K_M_* for the substrate.

To identify an endogenous inhibitor of Hmgcr, we took a candidate approach, focusing on ketone bodies first. We chose these metabolites because CoA thioesters of ketone bodies inhibit carbonic anhydrases *in vitro* (Parkkila et al., 2012) and β-hydroxybutyrate inhibits histone deacetylases *in vivo* (Shimazu et al., 2013). These metabolites also share a potential pharmacophore moiety with statin inhibitors of Hmgcr (Fig. 5A). Neither β-hydroxybutyryl-CoA nor acetoacetyl-CoA, however, inhibited human HMGCR *in vitro* with a physiologically plausible potency: the inhibition constant (*K_i_*) values for these metabolites were 10-and 14-times greater than the Michaelis-Menten constant (*K_M_*) for HMG-CoA, respectively (Fig. 5B). This lack of inhibition also comports with our observation that ketone bodies do not accumulate in *slc16a6a* livers: the carbon atoms are rerouted to other metabolites (Fig. 2F,G).

Interestingly, mice have lower serum cholesterol, hepatic cholesterol and hepatic Hmgcr activity when fed a defined diet with very high levels of a single PUFA (Du et al., 2003). In addition, intrahepatic fatty acyl-CoAs increase during fasting (Tubbs and Garland, 1964), and Hmgcr is inhibited by select fatty acyl-CoAs *in vitro* (Kuroda and Endo, 1976; Faas et al., 1977; Faas et al., 1978; Lehrer et al., 1981; Roitelman and Shechter, 1989). Detailed kinetic analyses, however, were not reported in some of these studies and, for short-chain fatty acyl-CoAs, the kinetic parameters do not support physiological inhibition. Fourth, total free fatty acids were increased in *slc16a6a* mutant livers (Fig. 2D,E). These observations led to the hypothesis that *slc16a6a* mutants accumulate fatty acyl chains and their CoA thioesters are inhibitors of Hmgcr.

**Fig. 6.**
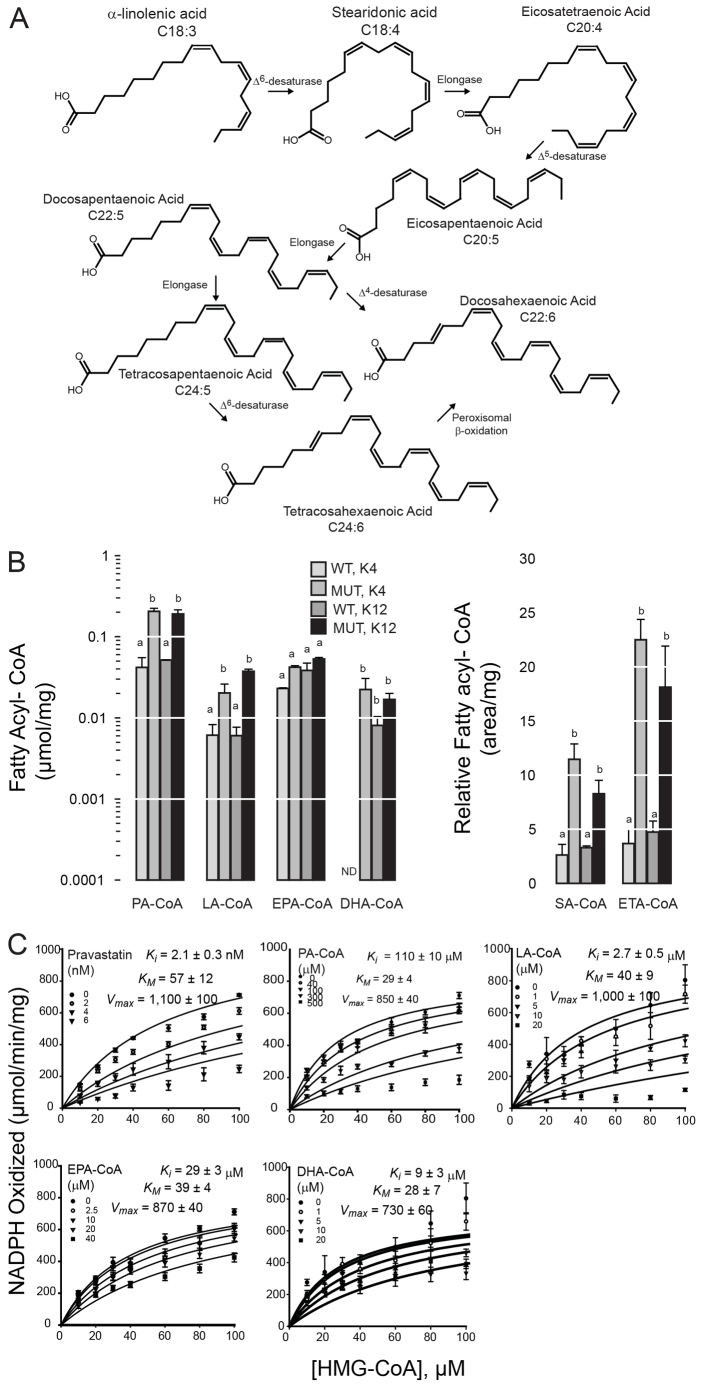
**PUFA-CoAs are competitive inhibitors of HMGCR.** (A) The synthetic pathway of n-3 polyunsaturated fatty acids is shown. From plant-derived α-linolenic acid (C18:3), a series of desaturation and elongation reactions generate two species that are the pharmacologically active species in fish oil preparations: eicosapentaenoic acid (C20:5) and docosahexaenoic acid (C22:6). The Δ^6^-desaturase that catalyzes the conversion of α-linolenic acid to stearidonic acid (C18:4) is strongly expressed in fish species, but not in higher vertebrates. (B) After 45 days on ketogenic diets (K4 or K12), livers were harvested from wild-type (WT) and *slc16a6a* mutant (MUT) livers, and fatty-acyl CoAs were analyzed. Abundance of individual species is normalized to protein mass (left). For stearidonyl-CoA (SA-CoA) and eicosatetraenyl-CoA (ETA-CoA) (right), commercial standards are not available, and results are reported as relative abundance (chromatogram area/mg protein). Groups with different letters are statistically different, *P*≤0.05, Tukey’s HSD. Data are mean ± s.e.m. (*n*=3 per group). PA, palmitoyl; LA, linolenyl; EPA, eisocapentaenoyl; DHA, docosahexaenoyl; SA, stearidonyl; ETA, eicosatetraenoyl. ND, not detected. (C) Michaelis-Menten analysis of human HMGCR in the presence of the indicated inhibitors.

We found that six FA-CoAs could be reliably detected in wild-type and *slc16a6a* mutant livers at the conclusion of the feeding study. Palmitoyl-CoA (PA-CoA) was the most abundant species and was increased in both *slc16a6a* groups. Linolenyl-CoA (LA-CoA) showed a similar increase in *slc16a6a* groups. The PUFA-CoAs eicosapentaenyl-CoA (EPA-CoA) and docosahexaenoyl-CoA (DHA-CoA) were not increased in the mutants (Fig. 6A); however, the intermediates of their synthesis, stearidonyl-CoA (SA-CoA) and eicosatetraenyl-CoA (ETA), were increased two- to three-fold, although the lack of commercial standards precluded absolute quantification of these two species.

Because several fatty acyl-CoA species were more abundant in *slc16a6a* livers, we tested whether they were inhibitors of human HMGCR in Michaelis-Menten analysis (Fig. 6B). We included EPA-CoA and DHA-CoA in these studies because they are the active components of pharmaceutical fish oils. We found that LA-CoA, EPA-CoA and DHA-CoA are physiologically plausible competitive HMGCR inhibitors (*K_i_* for each was less than the *K_M_* for HMG-CoA). Unsaturation seemed to be crucial to this inhibitory activity: *K_i_* of fully saturated PA-CoA is more than double the *K_M_* for HMG-CoA, yet its abundance is increased in *slc16a6a* mutant livers (Fig. 6A).

**Fig. 7.**
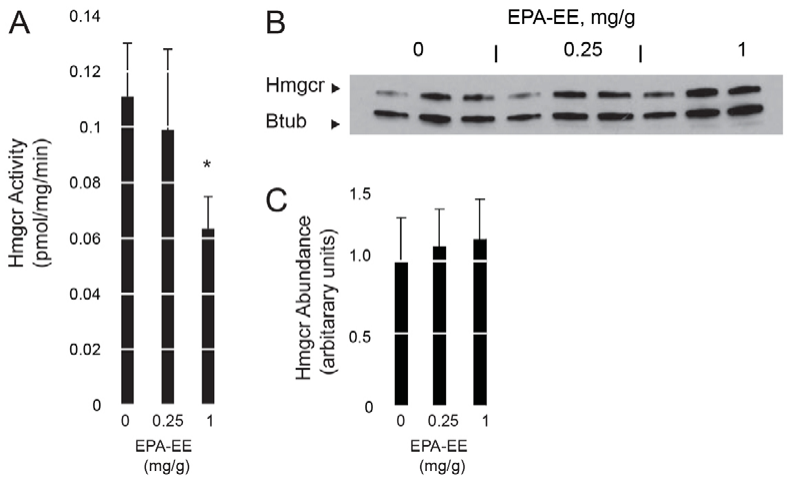
**Acute administration of a PUFA inhibits Hmgcr activity.** (A) Hmgcr activity in liver homogenates of wild-type mice following intra-peritoneal injection of EPA-ethyl ester (EPA-EE). *P=0.05, two-sided Student’s *t*-test. Data are means ± s.e.m. (*n*=3 per group). (B) Hmgcr protein abundance following EPA-EE injection (one mouse liver per lane). Btub, β-tubulin. (C) The relative abundance of Hmgcr protein in panel B was quantified by band densitometry, with normalization to the Btub signal. Results are shown in arbitrary units with the mean Hmgcr abundance for the vehicle-injected animals set to 1 ± s.d. (*n*=3). There was no difference in Hmgcr protein abundance among the three cohorts.

Although these *in vitro* findings were promising, the *K_i_* values for PUFA-CoAs are, nevertheless, higher than those of statin drugs. This suggests that inhibition of Hmgcr by PUFA-CoAs *in vivo* requires achieving relatively high intracellular concentrations. We directly tested whether acute administration of PUFAs might decrease Hmgcr activity *in vivo* by injecting mice intraperitoneally with EPA-ethyl ester (EPA-EE), a pharmacological form of an n-3 PUFA that is limiting in mammals (Huang et al., 1991). There was a decrease in hepatic Hmgcr activity observed with the higher dose of EPA-EE (Fig. 7A). Parallel injection of a statin inhibitor of Hmgcr caused a dose-dependent decrease in activity, as well (supplementary material Fig. S4A). Importantly, Hmgcr protein abundance was not altered by this short course of either EPA-EE or statin treatment (Fig. 7B,C and supplementary material Fig. S4B).

## DISCUSSION

Treatment of pandemic atherosclerosis poses a major therapeutic challenge in that the most effective agents – statin HMGCR inhibitors – are not uniformly tolerated. Furthermore, large adequately powered trials of statins in combination with a fibrate, niacin or inhibitors of cholesterol absorption have not demonstrated added benefit, despite enhanced lipid lowering (Baigent et al., 2011; Ginsberg et al., 2010; Boden et al., 2011). Furthermore, unanticipated side effects such as renal impairment (Bonds et al., 2012), and muscle and liver injury are observed in these trials (HPS2-THRIVE Collaborative Group, 2013).

Although our initial goal in this work was to address why blocking ketone body secretion triggers a triacylglycerol-only steatosis, we have uncovered a novel therapeutic possibility for overcoming the therapeutic shortcoming of statin adjuvants. By developing high-protein ketogenic diets, we were able to create experimental conditions for demonstrating that, when export of ketone bodies from the liver is impaired, PUFA-CoAs accumulate. Because zebrafish can synthesize PUFAs from plant-derived dietary unsaturated fatty acids (UFAs) (Hastings et al., 2001; Tocher et al., 2001), this model organism provided a unique opportunity to investigate the effect of PUFAs on cholesterol synthesis in a vertebrate. Our findings in this organism indicate that liver-trapped carbon atoms can accumulate into PUFAs that are not only incorporated into triacylglycerol, but their CoA thioesters inhibit cholesterol synthesis. Metabolite-based modulation of Hmgcr activity seems to occur in parallel to known direct regulators of this enzyme, such as phosphorylation or proteasome-mediated degradation (Burg and Espenshade, 2011).

Chronic PUFA supplementation also impairs Srebp1c maturation in mice, and thereby limits *de novo* lipogenesis. PUFA-mediated Srebp1c inhibition seems to involve two mechanisms. The first involves the altered biophysical properties of endoplasmic reticulum membranes when PUFAs are incorporated in phospholipid side chains: such incorporation into phospholipids suppresses the unfolded protein response, a potent trigger of Srebp1c activation (Ariyama et al., 2010; Lagace and Ridgway, 2013). The second mechanism of PUFA-mediated Srebp1c inhibition involves antagonism of Liver X receptor (Lxr)-driven induction of Srebp1c expression (Yoshikawa et al., 2002; Howell et al., 2009). These mechanisms governing Srebp1 regulation act in parallel to one another, and with still other known pathways. For instance, chronic fish oil supplementation triggers selective repression of mouse nuclear Srebp1c accumulation, and accelerates Srebp1c proteosomal degradation; interestingly, PUFA supplements seem to have no effect on Srebp2 (Botolin et al., 2006). Zebrafish seem to have only a single *srebf1* transcript variant, unlike higher vertebrates, in whom two major variants encoding Srebp1a and Srebp1c are present. Zebrafish have a single Lxrα ortholog, making this model organism potentially useful for examining Srebp regulation by Lxr in the future (Bertrand et al., 2007; Archer et al., 2008).

Thus, our *in vitro* studies with purified human HMGCR catalytic domains indicate that acute HMGCR inhibition by their CoA thioesters is another mode whereby PUFAs regulate lipid metabolism. PUFA-CoAs seem to be unique in their ability to inhibit HMGCR, in that fully saturated PA-CoA had physiologically implausible kinetic parameters. Likewise, the short course of EPA-EE decreased Hmgcr activity in mouse liver homogenates, without altering Hmgcr abundance. Thus, contrary to initial reports (Taketa and Pogell, 1966; Zahler et al., 1968) but consistent with later observations (Powell et al., 1981; Constantinides and Steim, 1985), fatty acyl-CoAs are not merely detergents that denature a broad range of enzymes.

Although feeding of high-DHA diets lowers serum cholesterol, reduces hepatic cholesterol content and lowers hepatic Hmgcr activity in mice (Du et al., 2003), conventional fish oil doses (rich in DHA and EPA) do not lower serum cholesterol in humans (Eslick et al., 2009). This contrasts with the low total cholesterol seen in native Greenlanders (Bang et al., 1971), a population that consumes diets very high in n-3 fatty acids and has a very low prevalence of coronary atherosclerosis (Bang et al., 1980). Moreover, diets high in PUFAs are associated with low levels of serum cholesterol and triacylglycerol in many populations, and with low incidence of atherosclerosis (Keys et al., 1966). Promisingly, high-dose supplementation with fish oils rich in DHA has a fairly potent serum-cholesterol-lowering effect even in the setting of statin-treated dyslipidemia, particularly if the total cholesterol remained high following statin initiation (Meyer et al., 2007). Likewise, trials of high-dose EPA report a similar cholesterol- and triacylglycerol-reducing effect, even when EPA is combined with a statin (Bays et al., 2011; Ballantyne et al., 2012). Crucially, a lower dose of fish oil supplementation does not confer cardiovascular protection (Roncaglioni et al., 2013).

In addition to these dose considerations, the formulation of fish oils affects serum cholesterol parameters. Conventional fish oil preparations (ethyl esters of PUFA mixtures) cause a striking increase in serum LDL cholesterol, often in proportion to the robust serum triacylglycerol lowering effect (Harris et al., 1997; Pownall et al., 1999). These effects seem to be related to the amount of DHA-EE relative to EPA-EE in the n-3 PUFA-EE formulations studied (Jacobson et al., 2012a), although this hypothesis has been challenged (Baum, 2012; Jacobson et al., 2012b). Newer formulations of PUFAs with greater oral bioavailability than PUFA-EEs have been prepared, and they seem to not raise LDL cholesterol (Maki et al., 2013). We found that both DHA-CoA and EPA-CoA inhibited human HMGCR *in vitro* with kinetic parameters consistent with *in vivo* relevance. Because the weight of clinical evidence points to EPA-EE as having LDL-cholesterol-lowering properties, we chose to administer this single species to mice. Recognizing that mice have extensive intestinal first-pass metabolism that differs substantially from humans (Komura and Iwaki, 2008), we administered EPA-EE intraperitoneally, and achieved a dose-dependent decrease in hepatic Hmgcr activity. These pharmacokinetic and pharmacodynamic concerns will, ultimately, be addressed only by hard outcomes clinical trials: a phase III mortality trial of pure EPA-EE (REDUCE-IT, NCT01492361) should provide these answers.

In summary, we have presented biochemical, physiological and pharmacological evidence that Hmgcr is inhibited by PUFA-CoAs. Our findings provide a mechanistic basis for the long-standing epidemiological observations that consumption of PUFA-rich diets is associated with low serum cholesterol and lower incidence of atherosclerotic cardiovascular disease. They also support developing and testing new formulations of PUFA supplements for lowering cholesterol.

## MATERIALS AND METHODS

### Experimental animals

These studies were approved by the University of Utah Institutional Animal Care and Use Committee and the Radiation Safety Committee. Zebrafish work was performed in the laboratory of A.S.; mouse experiments were performed by the laboratory of Donald A. McClain.

### Zebrafish

The *red moon^s951^* null allele of *slc16a6a* was as we reported previously (Hugo et al., 2012). It is carried on a WIK background, and the WT strain used as a control in all experiments is WIK.

### Diets

Two isoproteic and isocaloric diets differing in lipid content were formulated and prepared as explained previously (Karanth et al., 2009). For all feeding studies, sixty 30-day post-fertilization (dpf) homozygous *slc16a6a* mutant animals and sixty 30-dpf homozygous WT animals were distributed in 3 liter Aquatic Habitats tanks (ten fish per tank) and were fed defined diets for 45 days. Animals were housed in the main aquarium of the Centralized Zebrafish Animal Resource facility of the University of Utah and maintained on a 14-hour light, 10-hour darkness cycle. Animals were anesthetized by immersion in ice water (Wilson et al., 2009). For fasting studies, 4-month post-fertilization homozygous *slc16a6a* mutant animals and homozygous WT animals reared on conventional diet (commercial flakes and newly hatched *Artemia salina* nauplii, twice a day) were fasted for 10 days.

### Morphometric analysis and body composition

During the dietary study, animal mass was measured weekly. At the conclusion of the dietary study, animal length and mass were measured. Length is defined as the distance between the tip of the snout and the caudal peduncle, and was recorded using a Vernier caliper. The composition of fish samples was analyzed at the Hagerman Fish Culture Experimental Station, University of Idaho, following the Association of Official Analytical Chemists standards (Helrich, 1990). Ten fish per group were analyzed.

### Light and electron microscopy

Histological analysis was performed at AML Laboratories and transmission electron microscopy was performed at the University of Utah Electron Microscopy Facility, exactly as we described previously (Hugo et al., 2012).

### RNA isolation, cDNA synthesis and gene expression profiling

Total RNA was isolated from liver of zebrafish using the standard TRIzol method (Invitrogen, Carlsbad, CA). mRNA in 2 mg of total RNA was converted to cDNA using oligo(dT) primer and random hexamers according to the manufacturer’s instructions for the Clontech (Takara Biotech, Mountain View, CA). The primers for all the transcripts that we measured were described previously (Hugo et al., 2012). The target sequence for each gene was quantified to generate a standard curve of known copy number. Amplification of cDNA samples and DNA standards was carried out according to the manufacturer’s instructions. For thermal cycling and fluorescence detection, an Agilent Technologies Stratagene MX 3000P machine was used.

### Immunoblotting

Livers were sonicated in 400 μl of 50 mM Tris (pH 8.0), 150 mM NaCl, 1.0% IGEPAL CA-630, 0.25% sodium deoxycholate, 0.1% SDS and 1 mM EDTA supplemented with protease and phosphatase inhibitor cocktails (Roche Complete MINI and PhosSTOP). Twenty μg of protein (concentration determined with a BCA kit, Thermo) from each lysate was separated by SDS-PAGE, transferred to nitrocellulose membranes and detected with commercially available antibodies: Acaca IgGs were from Cell Signaling (3662, rabbit), Hmgcr IgGs were from Santacruz (H-300, rabbit), Srebf1 IgGs were from Active Motif (clone 2A4, mouse), Srebf2 IgGs were from Novus Biologicals (NBP1-71880, rabbit) and β-tubulin was from Abcam (ab6046, rabbit). ImageJ was used to quantify the relative density of protein bands. Following exposure, X-ray films of immunoblots were scanned. The images were inverted and the raw integral density was quantified for each band. Then the raw integral density of each Hmgcr band was divided by the raw integral density of the corresponding β-tubulin (Btub) band. The mean band density ratio for the vehicle-treated cohort (0 mg/g EPA-EE) was set to unity.

### Blood chemistry

Blood glucose levels were measured using a glucose meter (Contour, Bayer). Total lipids were extracted from serum, liver and whole body following the Folch method as described (Iverson et al., 2001). Total cholesterol, cholesterol ester, triglyceride and free fatty acids were resolved using thin layer chromatography (TLC) exactly as we described previously (Schlegel and Stainier, 2006). The abundance of each lipid class was quantified using a standard charring and copper-based densitometric assay (Bitman and Wood, 1982; Ruiz and Ochoa, 1997). Ketone bodies were quantified using a Hitachi HPLC equipped with a Capcell Pak 5 μm C8 UG 120 Å, LC Column (150×4.6 mm) as described in detail (Yamato et al., 2009). Briefly, liver lysates were mixed and incubated with the diazo reagent and introduced to HPLC to quantify the acetoacetate. 3-hydroxybutyrate in the liver lysate was enzymatically converted to acetoacetate and quantified by comparing to the standard curve.

### Hmgcr activity assays

Livers were removed, washed in 0.9% NaCl, and homogenized in 5 volumes of 100 mM sucrose, 50 mM KCl, 40 mM KH_2_PO_4_, 30 mM EDTA, pH 7.4, with a tissue grinder. After centrifugation at 12,000 ***g*** for 15 minutes to remove insoluble debris, Hmgcr activity was assayed as described previously by monitoring the conversion of ^14^C-3-hydroxy-3-methlyglutaryl-CoA to ^14^C-mevalonate, using ^3^H-mevalonolactone as an internal recovery standard (Erickson and Fielding, 1986). For studies of purified human HMGCR, oxidation of NADPH was monitored colorimetrically in the presence of a series of potential inhibitors. The assays were conducted in 96-well plates. Each well contained 200 μl of assay mixture (buffer and HMG-CoA, Sigma). The buffer consisted of 600 mM KCl, 1 mM EDTA, 100 mM KH_2_PO_4_, 1 mg/ml BSA, 200 mM NADPH, and 3 mg/ml human HMGCR, pH 7.3 (Bischoff and Rodwell, 1996). The assay mixture was dispensed to all the wells and varying concentrations of inhibitor were added. HMG-CoA was added to start the reaction. Extinction of NADPH was measured at 340 nm every 30 seconds for 10 minutes using a Biotek plate reader. Each assay was performed in quintuplicate. The Enzyme Kinetics module in SigmaPlot 12.3 (Systat Software, San Jose, CA) was used to analyze kinetic data. Errors are automatically propagated with this software and kinetic parameters (*V_max_*, *K_M_* and *K_i_*) are reported as mean ± standard deviation.

### Radiotracer studies

2.5-month-old *slc16a6a* mutant and WT zebrafish fed defined diets for 40 days were intraperitoneally injected with 5 μl of ^3^H-acetate [1.3×10^6^ counts per minute (CPM)] or ^3^H-mevalonolactone (0.8×10^6^ CPM) at 8:00 pm (Kinkel et al., 2010). Twelve hours later, fish were euthanized and dissected. Total lipid from the livers were extracted and dissolved in 200 μl of chloroform, 50 μl of which was loaded onto the TLC plates. After the TLC plates were developed, the plates were placed in a chamber saturated with iodide vapor to visualize resolved lipid species. The cholesterol (free and esterified) and triacylglycerol fractions were individually scraped off and put in scintillation vials. Four ml of scintillation fluid was added to the vials and vortexed vigorously. Vials were then placed in the scintillation counter to measure disintegrations. In parallel, 50 μl of the lipid extract was resolved on TLC plates, and abundance was quantified with copper impregnation and charring. For the fasting radiotracer study, 4-month-old homozygous *slc16a6a* mutant and homozygous WT animals reared on the facility diet were fasted for 10 days, and were then injected intraperitoneally with 5 μl of ^3^H-acetate (1.3×10^6^ CPM). The following morning, livers were harvested 1 hour after a second injection of EPA-EE or vehicle, and subjected to lipid extraction. TLC was performed in duplicate, with half of each sample run for the purpose of scrapping the plates and performing scintillation counting on individual bands, and the other half run on a plate that was subsequently charred for quantification of lipid species mass.

### Fatty acyl profiling

Fatty acid methyl esters were prepared as described in detail (Li and Watkins, 2001), separated by gas chromatography and analyzed by mass spectrometry at the University of Utah Metabolomics Core Facility. Analysis was performed with a Waters GCT Premier mass spectrometer fitted with an Agilent 6890 gas chromatograph on a 30 m Phenomenex ZB5MSi column with a 5 m long guard column.

### Fatty acyl-CoA profiling

Fatty acyl-CoAs were extracted from pooled livers (*n*=3–5) by following the protocol described (Minkler et al., 2008). Livers were homogenized in acetonitrile/isopropanol (3 + 1, *v* + *v*), and centrifuged after adding 0.1 M KH_2_PO_4_. The supernatant was filtered through a preconditioned 2-(2-pyridyl) ethyl-functionalized silica gel SPE column (Sigma-Aldrich # 54127-U). The column was washed with 1 ml of acetonitrile/isopropanol/water/acetic acid (9 + 3 + 4 + 4, *v* + *v* + *v* + *v*) and the fatty acyl-CoAs were eluted with 0.5 ml of methanol/250 mM ammonium formate (4 + 1, *v* + *v*). The elutant was analyzed using a capillary C18-HPLC column (0.3×100 mm) coupled to an electrospray ionization time-of-flight mass spectrometer (MS) (Bruker Daltonics, USA). The samples were eluted from the capillary C18 column with a linear gradient of 15–90% acetonitrile containing 0.05% dibutylamine at a flow rate of 5 μl/minute for 30 minutes. The MS was acquired in the negative ion mode at the following conditions: cone gas flow rate at 50 l/hour, nozzle temperature at 130°C, drying gas (N_2_) flow rate at 450 l/hour, spray tip potential at 2.3 kV and nozzle potential at 35 V.

### Mouse studies

Six-week-old male C57BL/6 mice were injected intraperitoneally with pravastatin or EPA-EE at 4:00 pm on the day prior to analysis and at 8:00 am on the day of analysis (*n*=3 mice per dose). The vehicle (control) was DMSO and the total volume delivered was 0.4 ml. Livers were harvested and whole-liver homogenate was used to measure the Hmgcr activity 1 hour after the second injection, as described above for zebrafish livers.

### Statistics

Microsoft Excel 2010, Sigmaplot 12.3 and IBM-SPSS 20.0 were used for statistical analysis. The significance level was set at *P*<0.05. The differences between the means were analyzed either by one-way ANOVA or two-tailed *t*-test. After ANOVA, post hoc comparisons were conducted using the Tukey’s HSD or Duncan’s multiple range test.

## Supplementary Material

Supplementary Material
