# Supplementary material for: Polyunsaturated fatty acyl-coenzyme As are inhibitors of cholesterol biosynthesis in zebrafish and mice
Source: Dis Model Mech. 2013 Sep 18;6(6):1365–77. doi: 10.1242/dmm.013425 (PMC3820260; doi:10.1242/dmm.013425)
Supplement: Supplementary Material [file supp_6_6_1365__index.html]

Polyunsaturated fatty acyl-coenzyme As are inhibitors of cholesterol biosynthesis in zebrafish and mice — Polyunsaturated fatty acyl-coenzyme As are inhibitors of cholesterol biosynthesis in zebrafish and mice — Supplementary Material 

# Polyunsaturated fatty acyl-coenzyme As are inhibitors of cholesterol biosynthesis in zebrafish and mice

## DMM013425 Supplementary Material

**Files in this Data Supplement:**

- **Supplementary Material PDF**
