# Supplementary material for: Polyunsaturated fatty acyl-coenzyme As are inhibitors of cholesterol biosynthesis in zebrafish and mice
Source: Dis Model Mech. 2013 Sep 18;6(6):1365–77. doi: 10.1242/dmm.013425 (PMC3820260; doi:10.1242/dmm.013425)
Supplement: Supplementary Material [file supp_013425_DMM013425.pdf]

Supplementary Materials

Supplementary Figures

Fig. S1 Full-sized electron micrographs from Figure 1B are shown.

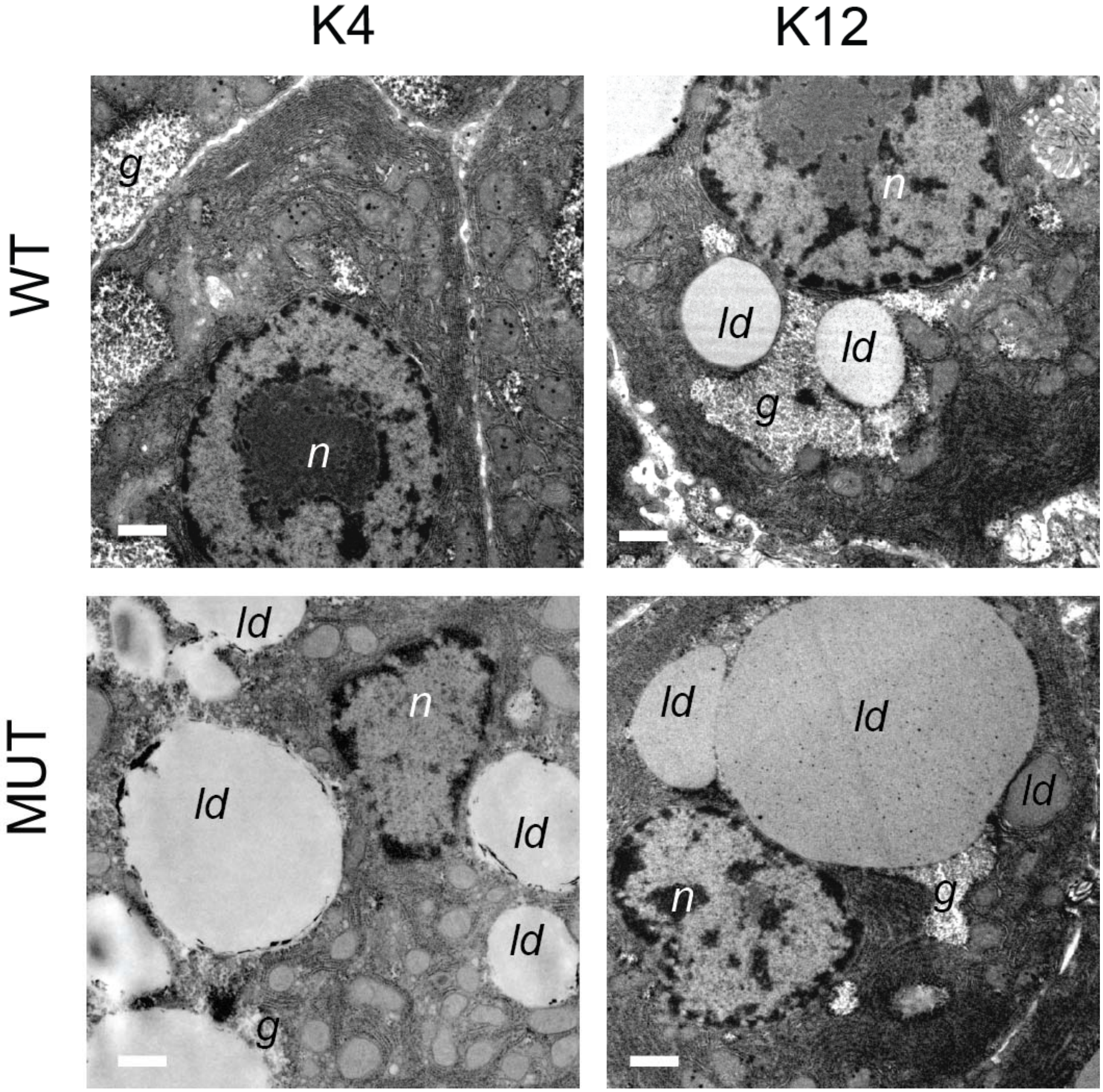

Fig. S2. *slc16a6a* mutant zebrafish gain weight faster than wildtype animals when placed on ketogenic diets. Wildtype (WT) and *slc16a6a* mutant (MUT) animals were fed the ketogenic diets (K4 and K12) described in Supplementary Table 2 for 45 days beginning 30 days post-fertilization. (**A**) Animals were weighed weekly (n = 10 per group). *$P < 0.05$, Student's t test. All data mean ± s.d. At the conclusion of the study, morphometric parameters were measured. (**B**) Mass, (**C**) length, and (**D**) Condition Factor (K), were greater in *rmn* mutants, and were independent of dietary fat content. WT animals showed greater mass, length and K on the higher fat diet (n = 6 for each group). $P < 0.05$ Tukey's HSD, with groups that are not statistically different from each other sharing a letter. All data are mean ± s.d.

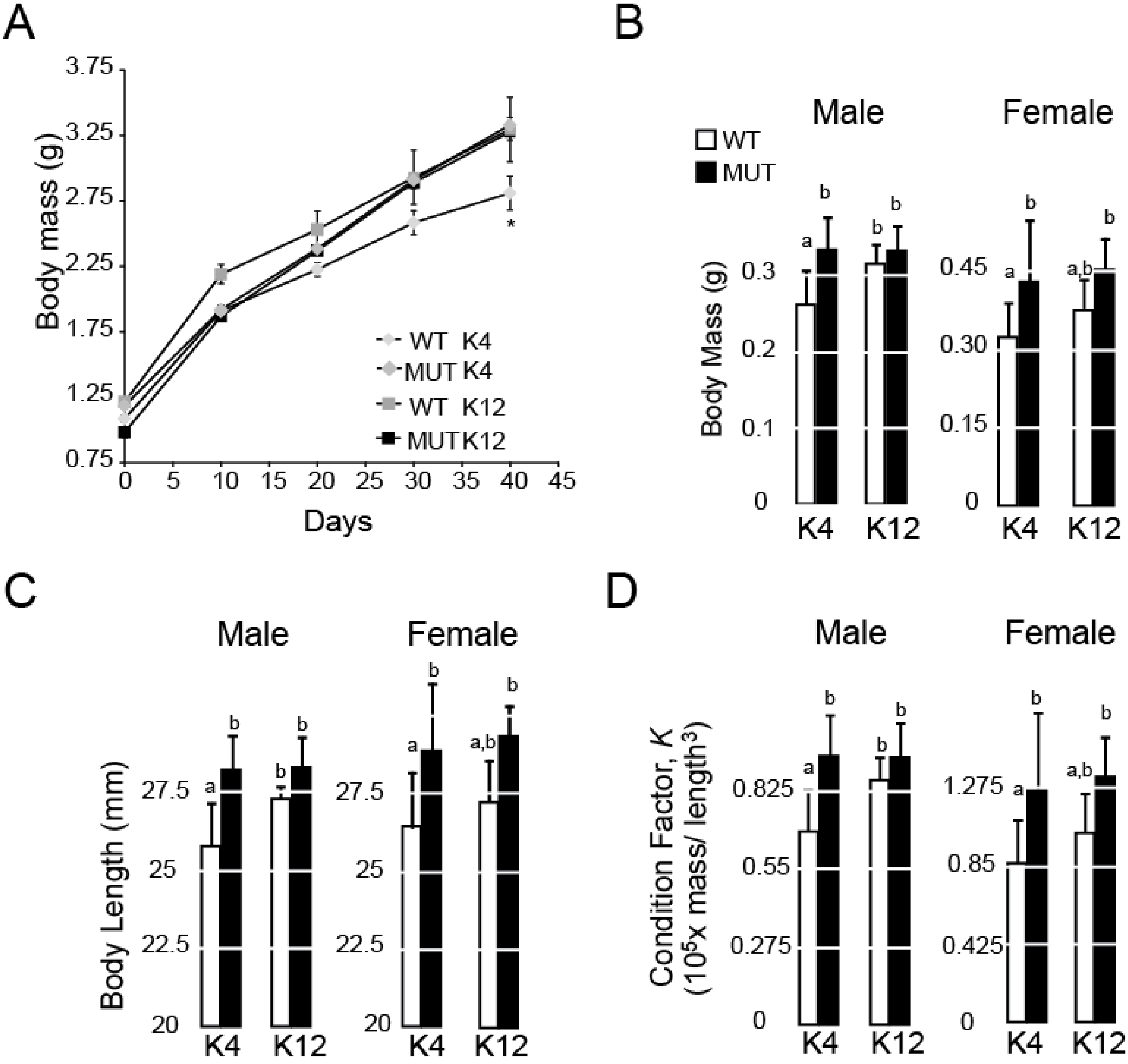

Fig. S3. Alignment of the phosphorylation site in select HMGCR orthologs. Human HMGCR is phosphorylated on Ser 872. The orthologous residues in Mouse Hmgcr (Ser 871), chicken HMGCR (Ser 872), zebrafish Hmgcr (Ser 868), and fruit fly Hmgcr are shown (Ser 898). The site is highlighted

```
H. sapiens      861 HLVKSHMIHN RSKINLGDLG GACTKKTA
M. musculus     860 HLVRSHMVHN RSKINLGDLG GTCTKKAA
G. gallus       861 HLVKSHMIHN RSKINLGDLG GTCTKKAA
D. rerio        857 HLVKSHMTHN RSKVNLQEAP GTCTKQAS
D. melanogaster 887 DLVKSHMRHN RSSIAVNSAN NPLNVTVSSCSTIS
```

Fig. S4. Pravastatin does inhibit mouse Hmgcr in vivo.(**A**) Following injection with vehicle or the indicated doses of pravastatin, mouse livers were harvested and assayed for Hmgcr activity. (**B**) Hmgcr protein abundance from each of the livers assaued in panel B was not affected by pravastatin injection. Btub, β-Tubulin.

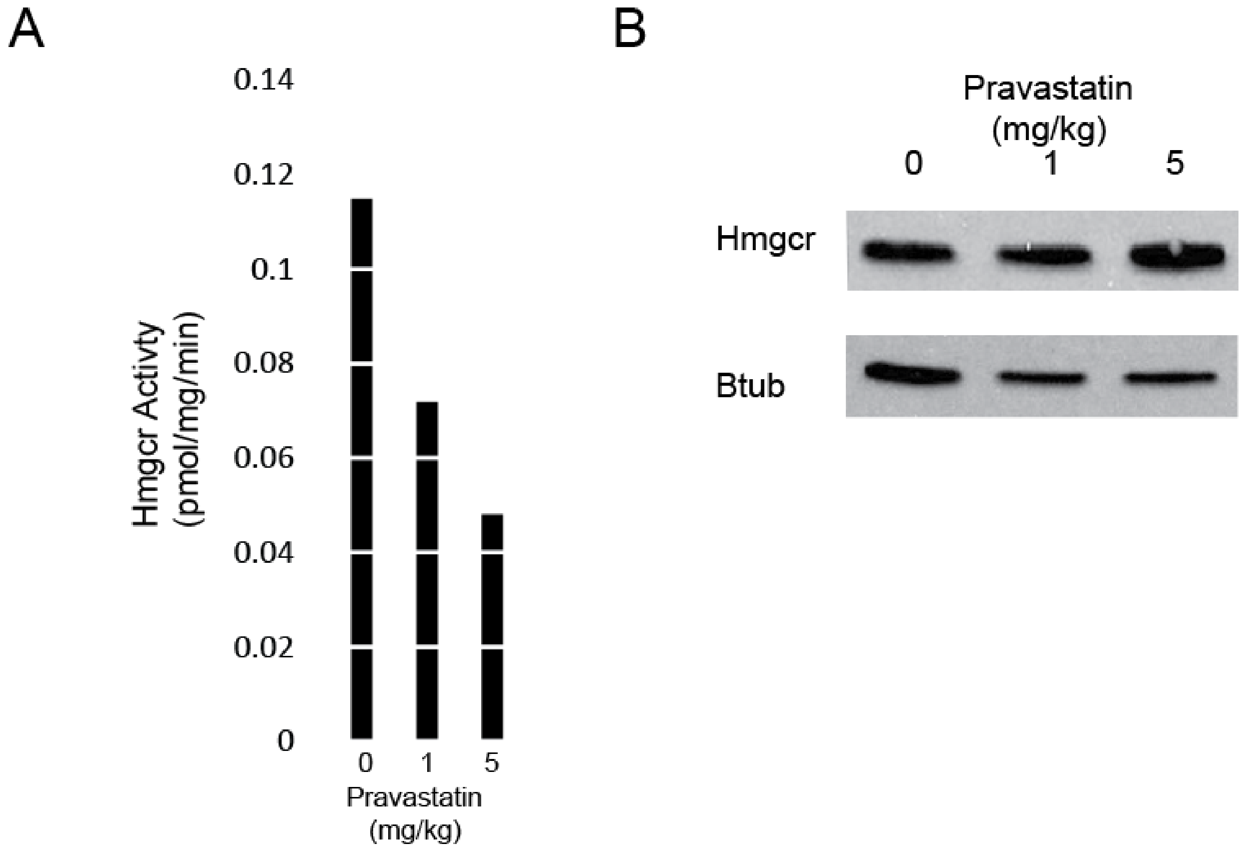

Supplementary Tables

Table S1. Defined, isocaloric, isoproteic, ketogenic diets used in the study. Leucine and lysine estimates are from Natinoal Research Council (1993) and Rombouts et al. (2009).

Ingredient (g/100 g diet)	K4 Diet	K12 Diet	Leucine (g)	Lysine (g)
Vitamin-free Casein	33	33	2.82	2.30
Wheat Gluten	10	10	0.72	0.14
Gelatin	4	4	0.11	0.14
Flax Oil	2	2		
Sunflower Oil	2	10		
Wheat Starch (pre-gelatinized)	33	25		
Celufil	8.5	8.5		
Choline Chloride	0.2	0.2		
Dicalcium Phosphate	5	5		
Stay C (vitamin C)	0.2	0.2		
Vitamin Mix[a]	0.8	0.8		
Mineral Mix[b]	0.1	0.1		
Betaine	1	1		
D,L-Methionine	0.2	0.2		
Total	100	100	3.65	2.58
DE (cal/g)	3,587.4	3,670.2		
**DE (MJ/kg)**	**15.2**	**15.4**		
BHT (preservative)	0.01	0.01		

a. Vitamin premix supplied the following per kg diet: vitamin A, 8,000 IU; vitamin D, 6,000 IU; vitamin E, 400 IU; vitamin K as menadione sodium bisulfite, 20 µg; thiamin as thiamin mononitrate, 32 mg; riboflavin, 64 mg; pyridoxine as pyridoxine-HCl, 64 mg; pantothenic acid as Ca-D-pantothenate, 192 mg; niacin as nicotinic acid, 240 mg; biotin, 0.56 mg; folic acid, 12 mg; vitamin B12, 50 µg; and inositol as meso inositol, 400 mg.
b. Composition of trace mineral premix (mg/kg premix): Zn (as $ZnSO_4.7H_2O$), 75; Mn (as $MnSO_4$), 20; Cu (as $CuSO_4.5H_2O$), 1.54; I (as KIO_3),10. The inclusion level of the mineral premix in the diet is 1g/kg diet. Composition of feed grade dicalcium phosphate (%, unles indicated otherwise): Calcium, 22; Phosphorus, 18.5; Sodium, 0.05; Potassium, 0.07; Magnesium, 0.6; Copper 80 mg/kg; Iron 10,000 mg/kg; Manganese 300 mg/kg; Selenium 0.6 mg/kg; Zinc 220 mg/kg.

Table S2. Comparison of macronutrient composition among the 2 defined diets used in this study and commercially available adult diets.

Ingredient (g/100 g diet)	K4 Diet	K12 Diet	Aquamax Grower 400®[a]	Nutrafin Max Flake Food®[b]	TetraMin Tropical Flakes®[c]
Protein	47	47	45	44	48
Fat	4	12	16	5	8
Fiber (Filler)	8.5	8.5	3	2	2

a. Purina Mills, LLC. Brentwood, MO.
b. Rolf C. Hagen Corporation, Mansfield, MA
c. Tetra Holding, Blacksburg, VA

Table S3. Body composition of wild-type (WT) and *slc16a6a* mutant (MUT) animals at the conclusion of the feeding study (n = 10 animals were pooled for analysis in each group).

	K4 Diet		K12 Didet	
	WT	*rmn*	WT	*rmn*
Water	75.1	71.6	73.8	71.5
Proteins	14.7	15.0	14.7	15.1
Ash	2.8	3.0	2.7	3.0
Lipids	7.5	10.6	8.2	10.9

Supplementary References

National Research Council (1993). Nutritional Requirements of Fish. Washington, DC: National Academic Press.

Rombouts, **I.**, **Lamberts**, **L.**, **Celus**, **I.**, **Lagrain**, **B.**, **Brijs**, **K. and Delcour**, **J. A.** (2009). Wheat gluten amino acid composition analysis by high-performance anionexchange chromatography with integrated pulsed amperometric detection. *J. Chromatogr. A* **1216**, 5557-5562.
